# Physicochemical and Interfacial Insights into Porphyrin-Loaded HPMC Hydrogels

**DOI:** 10.3390/gels12060514

**Published:** 2026-06-10

**Authors:** Rica Boscencu, Adina Magdalena Musuc, Mihai Anastasescu, Radu Petre Socoteanu, Andreea Mihaela Burloiu, Irina Atkinson, Raul-Augustin Mitran, Daniela C. Culita, Emma Adriana Ozon

**Affiliations:** 1 Faculty of Pharmacy, “Carol Davila” University of Medicine and Pharmacy, 6 Traian Vuia St., 020956 Bucharest, Romania; rica.boscencu@umfcd.ro (R.B.); andreea_burloiu@yahoo.com (A.M.B.); emma.budura@umfcd.ro (E.A.O.); 2 Institute of Physical Chemistry—Ilie Murgulescu, Romanian Academy, 202 Spl. Independentei, 060021 Bucharest, Romania; manastasescu@icf.ro (M.A.); s.radu@icf.ro (R.P.S.); iatkinson@icf.ro (I.A.); rmitran@icf.ro (R.-A.M.);

**Keywords:** hydroxypropyl methylcellulose hydrogels, polymer-porphyrin interactions, interfacial properties, structure-properties relationships, porphyrin photosensitizers

## Abstract

Hydroxypropyl methylcellulose hydrogels were designed as polymeric matrices for porphyrinic photosensitizer samples (5-(2-hydroxy-5-methoxyphenyl)-10,15,20-tris-(4-carboxymethylphenyl) porphyrin (P3.2) and 5,10,15,20-tetrakis-(4-carboxymethylphenyl) porphyrin (P3.1) to investigate their physicochemical behavior and structure–property relationships. Fourier transform infrared, UV–Vis, and fluorescence spectroscopy showed that both porphyrins remained monomerically dispersed in the polymeric matrix by establishing moderate interactions with HPMC by hydrogen bonding. X-ray diffraction and atomic force microscopy showed the uniform microstructural organization of the hydrogel matrix, while thermal analyses confirmed the stability of both studied systems. Rheological measurements demonstrated that the incorporation of porphyrins in the hydrogel network slightly modulates viscoelastic behavior. The swelling, density, and pH studies highlighted correlations between molecular interactions and macroscopic hydrogel properties. The swelling ratio determined after 6 h showed values of about 89% for the hydrogel of HPMC with P3.1. and about 92% for the hydrogel of HPMC with P3.2, respectively. The pH value was found to be 7.0 for both hydrogels. These results highlighted interfacial and physicochemical insights into polymer–porphyrin interactions in hydrogel matrices. All studies show that a controlled dispersion of chromophores preserves their monomeric state and controlled structure–property relationships.

## 1. Introduction

The effective management of malignant pathologies and the increasing prevalence of antimicrobial resistance constitute critical challenges in contemporary medical practice. Statistical data predict that at the European level, for the next 15 years, more than 10 million deaths per year will be caused by bacterial and fungal infections [1,2]. On the other hand, cancer remains one of the leading reasons for death worldwide, responsible for about 16.8% of global fatalities, and thus remains a key priority in public health and economic policy [3]. Both conditions present substantial medical challenges on a global scale and justify the need to identify effective strategies and the use of new compounds with ideal therapeutic potential and minimal contrary effects.

Photodynamic therapy (PDT) using porphyrins as photosensitizers represents a promising and versatile therapeutic strategy. Among its various applications, topical PDT stands out as a non-invasive strategy widely employed in the treatment of a range of cutaneous disorders [4,5]. The method involves the local application of a photosensitizer, followed by red light irradiation, leading to the production of reactive oxygen species (ROS) in the occurrence of molecular oxygen, resulting in cell death [5,6,7]. Moreover, PDT has demonstrated promising results not only in oncological dermatology but also in the treatment of bacterial and fungal infections, effectively inactivating a broad spectrum of pathogens, including antibiotic-resistant strains [8,9,10].

Porphyrin derivatives are currently one of the most frequently used photosensitizers (PS) in photodynamic therapy (PDT) due to their good selectivity for tumor cells and low cytotoxicity in the dark, associated with an excellent photophysical profile. These tetrapyrrole-type structures have a high molar absorption coefficient in the phototherapeutic window (600–800 nm), possess a high reactive oxygen species generation ability [5,6,7], and constitute the largest class of photosensitizers investigated in both clinical and preclinical studies [11]. Several pharmaceutical formulations incorporating tetrapyrrole-based PSs—such as Foscan^®^, Foslip^®^, Metvix^®^, Levulan^®^, Photogem^®^, and Purlytin^®^—are currently used in PDT applications [5,12].

Despite progress in the development of new photosensitizers, the use of porphyrin derivatives in PDT of cutaneous disorders remains limited due to their tendency to aggregate, which negatively affects tumor cell uptake [13].

To overcome these complications, several strategies have been advanced in the last few years [14,15,16,17]. This includes optimizing the architectural design of PS by attaching polar and nonpolar functional groups at the porphyrin ring in order to obtain an optimal hydrophilic/lipophilic ratio that can significantly decrease aggregation tendency and increase their cellular accumulation [18,19,20].

For this purpose, our research group has synthesized, through environmentally friendly and versatile methodologies, a series of A_3_B- and A_2_B_2_-type porphyrinic structures bearing various polar substituents (−OH, −OCOCH_3_, −COCH_3_) attached to the tetrapyrrolic core [15,21,22,23,24].

As an alternative strategy to structural modification, the use of hydrophilic polymers, including polyethylene glycol and cellulose derivatives, in the formulation of tetrapyrrole molecules enhances aqueous solubility, mitigates self-aggregation, and facilitates a controlled and site-specific release of the photosensitizer in tumor tissues [25,26,27,28].

Polysaccharide-based biopolymer hydrogels are versatile delivery systems in dermatology, owing to their nontoxicity, excellent biocompatibility, biodegradability, and capacity to preserve a moist environment that supports wound healing and tissue regeneration [29,30,31]. The incorporation of porphyrin-based photosensitizers into polysaccharide matrices may provide a controlled-release platform that improves the physicochemical stability and bioavailability of the photosensitizers, while enabling site-specific and sustained delivery for enhanced efficacy in topical photodynamic therapy.

Hydroxypropyl methylcellulose (HPMC) is widely employed in pharmaceutical formulations due to its distinctive physicochemical properties, high biocompatibility, and capacity to form stable hydrogel matrices. Its hydrophilic polymer network supports the sustained release of photosensitizers and improves porphyrin dispersibility by reducing molecular aggregation.

**Scheme 1:**
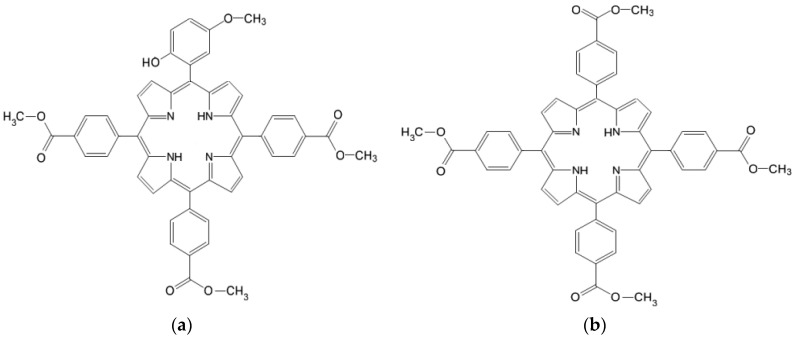
The structure of the investigated porphyrins: (**a**) 5-(2-hydroxy-5-methoxyphenyl)-10,15,20-tris-(4-carboxymethylphenyl) porphyrin (P3.2), (**b**) 5,10,15,20-tetrakis-(4-carboxymethylphenyl) porphyrin (P3.1).

In this study, the investigated system consists of hydroxypropyl methylcellulose (HPMC)-based hydrogels loaded with two structurally distinct porphyrinic photosensitizers (5-(2-hydroxy-5-methoxyphenyl)-10,15,20-tris-(4-carboxymethylphenyl) porphyrin (P3.2) and 5,10,15,20-tetrakis-(4-carboxymethylphenyl) porphyrin (P3.1) (Scheme 1)). HPMC is employed as a fixed and well-characterized hydrophilic polymer matrix, selected to ensure formulation stability, biocompatibility, and reproducibility, while allowing the isolation of the effects induced by the porphyrinic compounds. The study design is therefore centered on a porphyrin-dependent comparative approach, in which the polymeric carrier remains constant and the photosensitizer structure represents the main variable influencing the physicochemical and pharmacotechnical properties of the resulting hydrogels. This strategy enables a clearer understanding of how porphyrin structural features govern the behavior of topical hydrogel systems intended for photodynamic therapy applications.

These porphyrin–HPMC systems were subsequently physicochemically characterized using Fourier transform infrared spectroscopy (FTIR), X-ray diffraction (XRD), thermogravimetric analysis (TGA), atomic force microscopy (AFM), UV-visible, and fluorescence spectroscopy. Furthermore, pharmacotechnical evaluations were performed to assess the mechanical properties, pH, swelling ratio, and spreadability of the gels, aiming to establish the structure–property relationships and their suitability for applications in topical PDT in skin conditions.

Unlike previous studies primarily focused on the optimization of hydrogel matrices or the general incorporation of porphyrinic photosensitizers, the present work assumes a porphyrin-centered approach in which the polymeric carrier (HPMC) is intentionally kept constant as a validated and biocompatible delivery platform [32,33]. HPMC is a highly biocompatible cellulose used as a gelling agent in the concentration range of 1% to 4% [34]. HPMC was widely applied in topical formulation due to its ability to provide an excellent controlled release of active pharmaceutical ingredients and its excellent spreadability [35,36,37]. This research enables the systematic isolation and evaluation of the influence of porphyrinic molecular structure (different according to the upper aromatic substituent, Scheme 1) on the physicochemical and pharmacotechnical properties of the resulting hydrogels. By comparing two structurally distinct porphyrins (P3.1 and P3.2) under identical formulation conditions, the study provides direct evidence that the differences in porphyrin substitution patterns and polarity can significantly modulate hydrogel organization, swelling behavior, and surface characteristics. This approach offers new structure–property insights beyond conventional formulation studies and highlights the dominant role of photosensitizer chemistry in determining the performance of topical PDT systems.

## 2. Results and Discussion

### 2.1. Spectral Investigation of Polymer–Porphyrin Interactions

**Figure 1:**
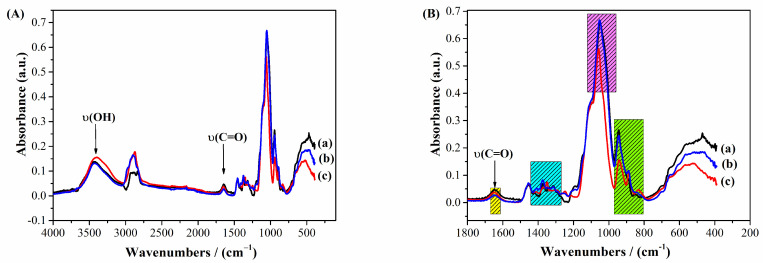
(**A**) FTIR spectra of HPMC-loaded porphyrin samples in the range of 4000–450 cm^−1^; (**B**) FTIR spectra of HPMC-loaded porphyrin samples in the range of 1800–450 cm^−1^; figure legend (a) HPMC (black color), (b) HPMC-P3.2 (blue color) and (c) HPMC-P3.1 (red color).

ATR-FTIR spectra of porphyrin-loaded hydrogels (HPMC–P3.1 and HPMC–P3.2) are shown in comparison with the ATR-FTIR spectrum of HPMC hydrogel (Figure 1A,B).

The FTIR spectrum of HPMC hydrogel (black color in Figure 1) exhibited characteristic bands for three spectral regions: (a) at ~3402 cm^−1^ (O–H stretching vibration), 2900 and 2828 cm^−1^ (C–H stretching vibration), (b) 1640 cm^−1^ (C=O bending), and (c) carbohydrate region at 1049 cm^−1^ (C–O–C stretching), which is in agreement with data in the literature [27,38]. The successful incorporation of porphyrins into the hydrogel matrix is supported by minor shifts observed in the O–H band (from 3430 cm^−1^ to 3418 cm^−1^ for HPMC-P3.1 (red color in Figure 1) and to 3433 cm^−1^ for HPMC-P3.2, (blue color in Figure 1), the sharpening and shifts in C–H band (from 2900 cm^−1^ to 2874 cm^−1^ for HPMC-P3.1 and to 2890 cm^−1^ for HPMC-P3.2), and the shifting and broadening of C=O band (from 1640 cm^−1^ to 1647 cm^−1^ for HPMC-P3.1 and to 1646 cm^−1^ for HPMC-P3.2). These changes indicate moderate polymer–porphyrin interactions through hydrogen bonding between the carboxyl and hydroxyl groups within the HPMC polymer matrix, while the other polymer bands remained unchanged, confirming the integrity of its structure. The structural feature of P3.1 and P3.2 porphyrins causes different modes of interaction with the specific bonds from the HPMC structure [39].

The FTIR analysis revealed only minor spectral shifts upon porphyrin incorporation into the HPMC hydrogel matrix, suggesting strong specific interactions such as hydrogen bonding. This observation is based on the presence of abundant –OH groups from the HPMC molecule that are involved in hydrogen bond formation with water and other molecules [35]. Therefore, the observed changes are more consistently interpreted in terms of a combination of weak non-covalent interactions, including electrostatic interactions and hydrophilic–hydrophobic balancing effects between the porphyrinic core and the HPMC network.

The differences observed between P3.1 and P3.2 formulations can be further correlated with their distinct substitution patterns, which influence molecular polarity and interaction propensity with the polymer chains. These structural variations reorganized the local environment of the porphyrins within the hydrogel, leading to minor but noticeable differences in vibrational bands (region marked in Figure 1b). The FTIR bands of P3.2 at 1610, 1505, 1434, and 1237 cm^−1^ and of P3.1 at 1610, 1505, 1434, and 1237 cm^−1^ are slightly shifted in the hydrogel matrix (patent application 201900799).

The absence of strong spectroscopic FTIR peaks specific to porphyrin suggests that the aggregation process is likely limited within the HPMC matrix. This behavior can be attributed to the relatively low loading concentrations, steric hindrance imposed by the hydrated polymer network, and favorable dispersion of porphyrinic species within the hydrophilic HPMC environment. Consequently, the hydrogel system appears to promote a predominantly molecularly dispersed state rather than extended π–π stacking interactions [40]. The hydrogen bonds are favored by the hydroxyl groups of HPMC and the oxygen-containing functional groups of the porphyrin. This leads to a shift in and broadening of the O-H stretching band, as evidenced by FTIR spectra from Figure 1.

Overall, the FTIR results support a model in which porphyrin–polymer interactions are governed primarily by weak electrostatic and polarity-driven effects rather than strong aggregation.

Porphyrins have attracted considerable interest for their potential use in PDT and antimicrobial photodynamic therapy (aPDT), owing to their distinctive structural and spectral characteristics. The spectral properties grant these structures the ability to generate highly cytotoxic reactive oxygen species (ROS) upon activation with laser light, offering potential for the treatment of various skin diseases [41,42]. Porphyrins exhibit a characteristic absorption spectrum consisting of a Soret band around 400 nm and four Q bands spanning the 440–700 nm range [43,44,45]. Although the Soret band shows much higher absorption intensity, the Q-band region is of primary relevance for photodynamic therapy applied to malignant and premalignant skin lesions, as photosensitizer activation occurs under laser irradiation within the 580–700 nm range. Moreover, the molecular structure, and specifically the aromatic character conferred by the π-electron system of the porphyrin ring, endows porphyrins with the ability to emit fluorescence within the 600–800 nm spectral range, thereby rendering them effective imaging agents.

UV–vis spectroscopy is frequently used to confirm the presence of porphyrins in the polymeric matrix through the identification of its absorption Soret and Q bands. Minor shifts in the absorption maxima by loaded porphyrin in the polymeric matrix are consequences of the hydrogen bonds that are established between the PS molecules and the polymer matrix.

**Figure 2:**
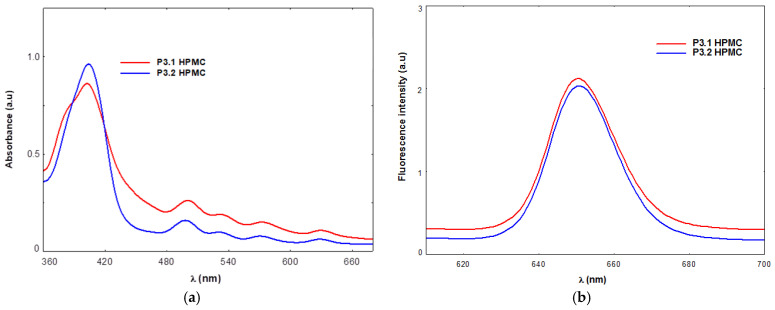
(**a**) UV-Vis spectra of P3.2. (blue) and P3.1. (red) samples in HPMC; (**b**) fluorescence spectra of P3.2 (blue) and P3.1 (red) in HPMC.

The spectral profiles of P3.2 and P3.1 porphyrins loaded into the HPMC matrix are presented in Figure 2, and the parameters associated with their absorption and emission spectra are summarized in Table 1.

The experimental data indicate the maintenance of the spectral profile of the two porphyrins included in the HPMC matrix, with absorption and emission maxima in the spectral range required for PDT [17]. Slight differences in the absorption maxima were evidenced (Table 1), reflecting structural variations between the two molecules and their interaction with the polymer matrix. In the polymeric matrix, the Q bands of P3.1 registered as slightly red-shifted compared to P3.2, due to differences in the substituents on the porphyrinic macrocycle. The presence of the O–H group in the structure of P3.2 promotes hydrogen bonding with the polymer matrix, leading to a slight blue shift and reduced absorption intensity compared to P3.1. For both hydrogels, the absorption spectra confirm the stability of the two porphyrins in this type of pharmaceutical formula, with all the relevant spectral characteristics remaining unaltered (Figure 2a). Also, no H- and J-aggregates are observed; the absorption spectra of the two porphyrins loaded with HPMC retained their characteristic sharp Soret and Q bands as evidence of their monomeric dispersion.

Regarding the fluorescence profile of the P3.1 and P3.2 loaded in the polymeric matrix (Figure 2b), the emission spectra of the two porphyrins kept the spectral profile typical of the free base porphyrins, with a decrease in fluorescence for P3.2 compared with P3.1 (Table 1), due to the electronic effects dictated by the structural particularities of the unsymmetrical porphyrin. The excitation at the same wavelength triggered similar emission responses for both gel samples. The signal of fluorescence registered is strong, proving that the interaction of porphyrin with the HPMC matrix has an insignificant influence on the amount of P3.1. and P3.2. in the fluorescence profile. The chromophores are stabilized through non-covalent interactions with the polymer network. In conclusion, under the experimental conditions investigated, both porphyrins exhibited absorption in the phototherapeutic range relevant to photodynamic therapy. Moreover, their emission spectra displayed the typical profile associated with porphyrinic photosensitizers with no aggregation, and slight shifts in the emission maxima were observed for the unsymmetrical porphyrin. The compounds P3.2 and P3.1, when incorporated into HPMC, showed strong fluorescence, which represents a significant advantage for their biomedical applications, by preserving their photophysical properties.

### 2.2. Structural Characterization

**Figure 3:**
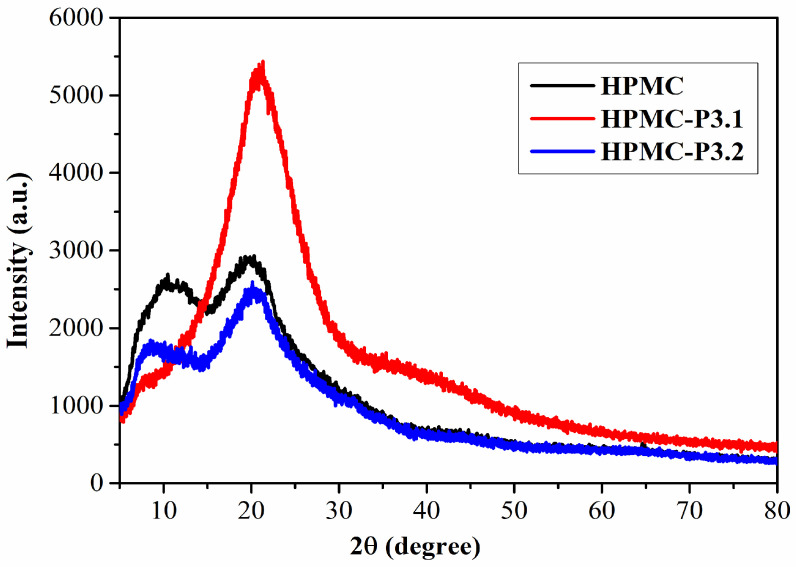
XRD diffractograms of HPMC, HPMC-P3.1, and HPMC-P3.2.

The XRD spectra of HPMC hydrogel and porphyrin-loaded hydrogels are shown in Figure 3.

X-ray diffraction spectrum of pure HPMC (black line in Figure 3) shows two broad peaks at 2θ = 10.24° and 20.3°, consistent with the amorphous character of the HPMC polymer [46]. For the HPMC-P3.1 sample, the first peak from XRD spectrum (red line from Figure 3) shows a small shift at 2θ~8.32° and a decrease in its intensity, while the second peak at 2θ~20.82° showed a significant increase in its intensity compared to pure HPMC, suggesting a short range enhancement in the hydrogel-based polymer network, likely resulting from interactions between HPMC chains and the incorporated P3.1 porphyrin, especially due to hydrogen bonding [47]. For the HPMC-P3.2 sample, the two peaks appeared at 2θ~8.3° and ~20.3° (blue line from Figure 3), but their intensities were slightly lower than in pure HPMC. This typically suggests that P3.2 porphyrin was uniformly dispersed into the hydrogel matrix by maintaining its amorphous character without promoting additional local ordering. The XRD analysis establishes that while porphyrin incorporation can modify the degree of local polymer ordering, the amorphous structure of the HPMC hydrogel matrix is almost well-maintained in both samples, especially for HPMC-P3.2. This evidence is also correlated to the FTIR findings, where small shifts in the O-H and C=O bands were observed.

**Figure 4:**
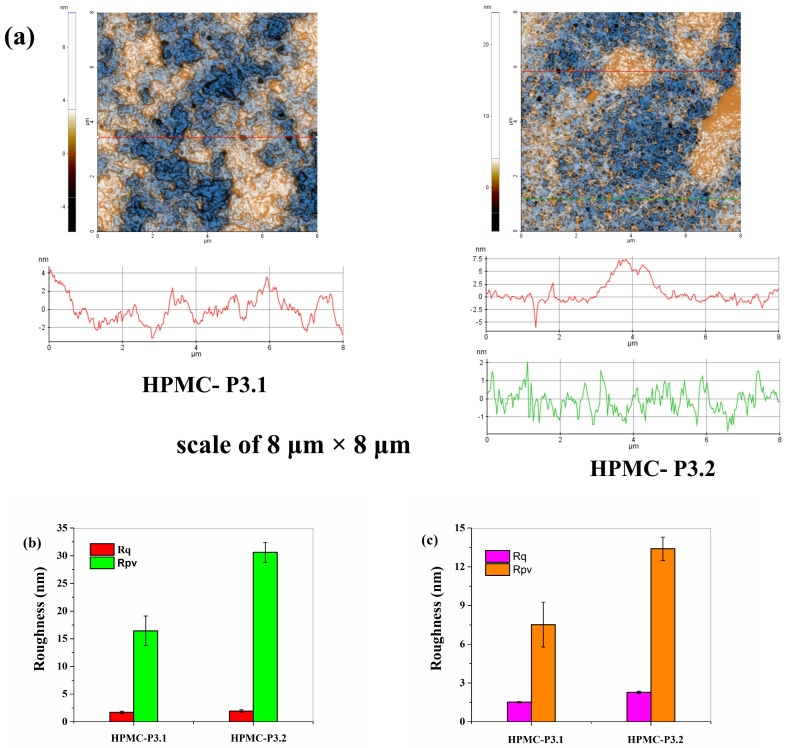
2D-AFM images and their characteristic line scans (marked with horizontal red and green lines) at the scale of (**a**) (8 × 8) μm^2^. Roughness (Rq) and peak-to-valley (Rpv) over the entire scanned areas: (**b**) (8 × 8) μm^2^, respectively, along the line scans over (**c**) for 8 μm (analyzed sample: HPMC-P3.1 and HPMC-P3.2).

**Figure 5:**
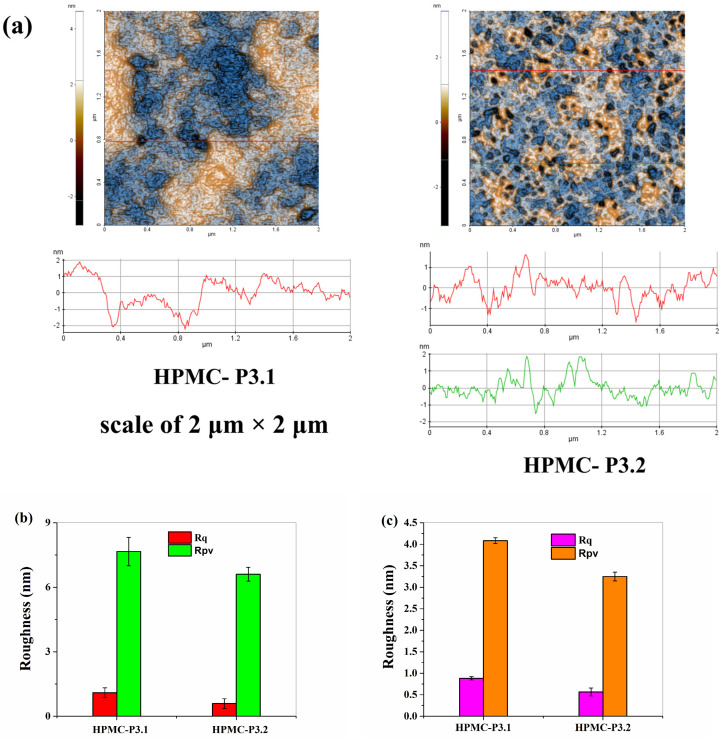
2D-AFM images and their characteristic line scans (marked with horizontal red and green lines) at the scale of (**a**) (2 × 2) μm^2^. Roughness (Rq) and peak-to-valley (Rpv) over the entire scanned areas: (**b**) (2 × 2) μm^2^, respectively, along the line scans over (**c**) for 2 μm (analyzed sample: HPMC-P3.1 and HPMC-P3.2).

The AFM images of the two samples (HPMC-P3.1 and HPMC-P3.2) are shown in Figure 4 and Figure 5, recorded with enhanced contrast at a scale of (8 × 8) μm^2^ (Figure 4) and at a smaller scale of (2 × 2) μm^2^ (Figure 5). The characteristic surface profiles, indicated by the corresponding line scans, are represented by the horizontal red and green lines, which show different topography at the nanoscale structure.

The AFM images at a larger scanning area (8 × 8) μm^2^ (Figure 4a) show a relatively homogeneous surface for both samples (HPMC–P3.1 and HPMC–P3.2) characterized by low-amplitude undulations and a continuous polymer network. No appearance of pronounced domains or large segregated structures was observed, suggesting a uniform distribution of porphyrins within the HPMC matrix. The surface corrugation parameters calculated over the entire scanned area indicate a moderate roughness, reflecting the intrinsic morphology of the polymer hydrogel. For the whole area, the sample HPMC–P3.1 is characterized by an RMS roughness (Rq) of 1.6 nm and a peak-to-valley (Rpv) parameter of 16.4 nm, and for HPMC–P3.2, the Rq is 1.9 nm and Rpv is 30.6 nm (Figure 4b). These values indicated that both samples have surfaces that are very smooth at the nanoscale, although HPMC-P3.2 is slightly rougher than HPMC-P3.1. Nevertheless, the roughness values remain within the nanometric range, indicating the uniform, homogeneous morphology of the surface without large aggregates or phase-separated domains. The roughness (Rq) and peak-to-valley (Rpv) parameters along the line scans over 8 μm show the same profile as those from the whole area (Figure 4c).

At a smaller scanning scale, at (2 × 2) μm^2^ (Figure 5a), fine nanoscale surface features, including shallow depressions and small protuberances, are more clearly highlighted and were observed to be distributed across the surface for both samples. These structures are characteristic of the hydrated polymer networks and are likely due to the rearrangement of polymer chains during gel formation and drying. The roughness (Rq) and peak-to-valley (Rpv) parameters calculated for the whole area (2 × 2) μm^2^ (Figure 5b), and along a selected profile line (for 2 μm, Figure 5c), showed only moderate variations between the analyzed domains, indicating a relatively uniform surface organization. The peak-to-valley parameters obtained from the profile lines are almost the same for the whole area for both samples (for HPMC–P3.1, the Rq is about 1.1 nm, and Rpv is 7.6 nm, and for HPMC–P3.2, the Rq is about 0.6 nm, and Rpv is 6.6 nm), confirming that the differences remain within the nanometric range.

It can be concluded that on the AFM images, the presence of large aggregates is not observed, indicating that both porphyrin samples are homogeneously dispersed within the hydrogel network at the nanoscale level. This observation is corroborated by the spectroscopic data obtained from UV–Vis absorption and fluorescence measurements, which suggested that the porphyrins remain predominantly in a monomeric state after incorporation.

### 2.3. Thermal Analysis

**Figure 6:**
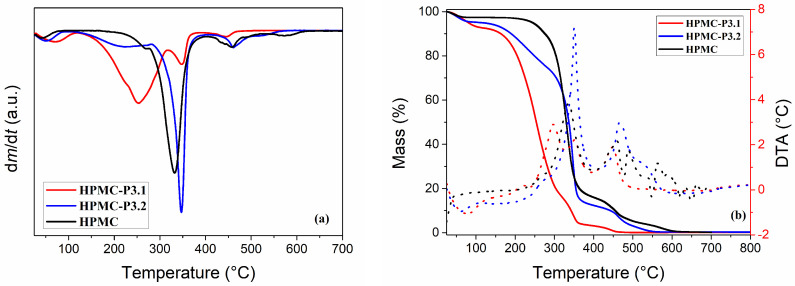
(**a**) DTG curves and (**b**) thermogravimetric (TG) (full lines) and differential thermal analyses (DTA) (dotted lines) of HPMC, HPMC-P3.1, and HPMC-P3.2 samples.

Thermogravimetric analyses (TGA) were carried out on the two samples (Figure 6a,b). The HPMC hydrogel exhibits ~2.5 wt.% mass loss on heating up to 100 °C and HPMC-P3.1 exhibits a 7.1 wt.% mass loss on heating up to 110 °C, while the HPMC-P3.2 sample contains 4.9% residual water. This process corresponds to a small endothermal peak on the DTA curve (Figure 6b). All samples decompose exothermically in three distinct steps, between 125 and 350, 350 and 450, and 450 and 600 °C. These temperature ranges correspond to the combustion of the organic groups with an exothermal effect on the DTA curve. Sample HPMC-P3.1 shows the greatest mass loss between 125 and 350 °C, while sample HPMC-P3.2 presents the highest mass loss between 350 and 450 °C.

Small variations in mass loss and the onset of degradation temperatures were observed, which were attributed to non-covalent polymer–porphyrin interactions within the hydrogel matrix, as evidenced by FTIR and XRD analyses. The maintenance of the main degradation steps highlights that the structural integrity and thermal stability of the HPMC network are maintained after porphyrin incorporation.

### 2.4. Swelling and Pharmacotehnical Properties

The organoleptic properties (such as color, appearance, homogeneity, consistency, pH, density, and presence of phase separation or any agglomerations) were evaluated according to the literature [48,49] and are presented in Table 2.

The prepared porphyrin-loaded hydrogels are translucent with a homogeneous structure without air bubbles, and no aggregation or phase separation occurs. These findings are also evidenced by AFM, UV–Vis, and fluorescence studies, which confirm the uniformity and consistent nanostructures of the samples. The density values are approximately close to each other for the two studied samples and are close to that of water (1.0 g/mL), being typical for hydrogels. These values indicate the hydration of the polymer hydrogel structure and the uniform dispersion of porphyrins. The similar values show that the type of porphyrin does not significantly modify the volumetric structure of the HPMC hydrogel, with no aggregation or phase separation occurring. The pH of the porphyrin-loaded HPMC hydrogel formulations was found to be 7.0, as shown in Table 3, indicating a nearly neutral character and a good compatibility with the skin [50]. Although the physiological pH of healthy skin is slightly acidic, a neutral pH is generally considered non-irritating and suitable for topical applications [51]. Also, studies in the literature demonstrate that an increase in the pH values of the hydrogel influences the swelling process and mechanical and thermal properties of its structure [52]. The most appropriate mechanical features are obtained at neutral pH [53].

The pharmacotechnical parameters of the tested HPMC-P3.1 and HPMC-P3.2 dried gels at room temperature are displayed in Table 3.

The evaluated porphyrin-loaded hydrogel films have a thickness of approximately 0.059 ± 0.002 mm for HPMC-P3.1 and of approximately 0.062 ± 0.003 mm for HPMC-P3.2. These values are aligned with the typical thickness of developed hydrogels for biomedical applications and demonstrate that a good hydrogel must have a thickness lower than 2 mm in order to be adhesive and stretchable. The folding endurance values were above 300 for both types of films. This parameter allows us to demonstrate the flexibility of each developed formulation. The obtained values are good compared with the literature and demonstrate that the hydrogels are not brittle [54]. In vitro adhesion ability was measured on dry gels. The elasticity, stretchability, and robustness of the hydrogels were evaluated by measuring the tensile strength. The registered tensile strength values were 1.02 ± 0.06 kg/mm^2^ for HPMC-P3.1 and 1.16 ± 0.23 kg/mm^2^ for HPMC-P3.2. The elongation values of the porphyrin-loaded HPMC gel are 20.13 ± 1.48% for HPMC-P3.1 and 26.05 ± 1.04% for HPMC-P3.2. These parameters are in good agreement with other developed hydrogels designed for biomedical applications, which demonstrated their favorable elasticity and stretchability to be applied on the skin [55].

**Figure 7:**
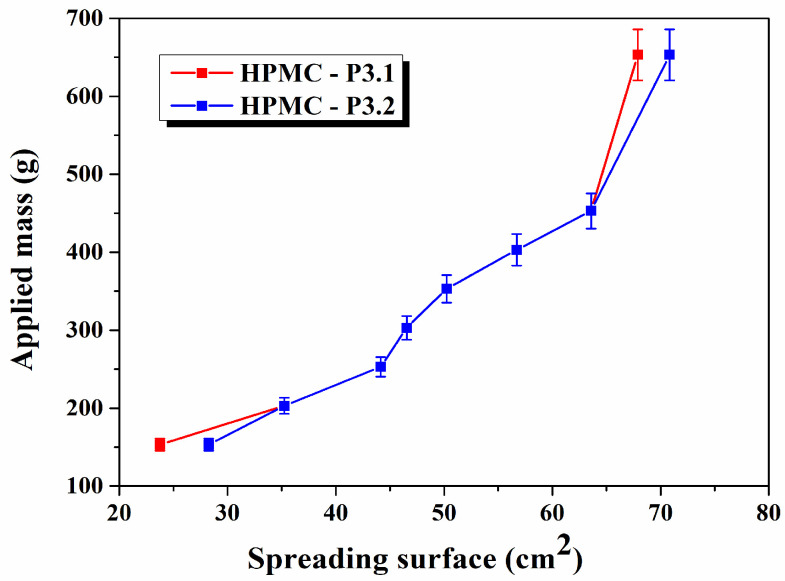
The spreading surface areas of HPMC-P3.1 (red line) and HPMC-P3.2 (blue line).

The spreading surface areas of the two porphyrin-loaded hydrogels are represented in Figure 7.

It was observed that the spreading surface (measured as the diameter of the produced circle when a weight is applied) varies and increases with the applied mass growth. The therapeutic effect of the hydrogel depends on the spread when it is applied to the skin.

From Figure 7, it was observed that the two samples exhibit similar behavior over the applied weight range of 203–453 g. A clear increase in the spreading surface was noticed up to 253 g for both samples, indicating a progressive deformation of the HPMC-P3.1 and HPMC-P3.2 network under compression force.

**Figure 8:**
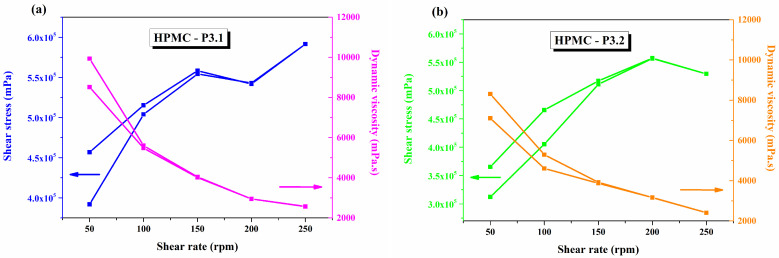
The rheological analysis of (**a**) HPMC-P3.1 and (**b**) HPMC-P3.2.

Rheological analysis is represented in Figure 8a for HPMC-P3.1 and Figure 8b for HPMC-P3.2. Shear stress curves for HPMC-P3.1 and HPMC-P3.2 demonstrate that the two samples exhibited shear-rate-dependent behavior. From the dynamic viscosity curves reported in Figure 8a,b, a pseudoplastic behavior is reported, as well as a disruption of weak intermolecular association by increasing the shear rate. When the shear rate increases, the shear stress increases and the dynamic viscosity decreases.

The two samples exhibit similar rheological behavior, except at a shear rate of 200 rpm, where the shear stress decreases for HPMC-P3.1 and then increases again at 250 rpm, whereas for HPMC-P3.2, the shear stress decreases at 250 rpm. These variations may reflect minor differences in the microstructural organization of the polymer network after porphyrin incorporation.

**Figure 9:**
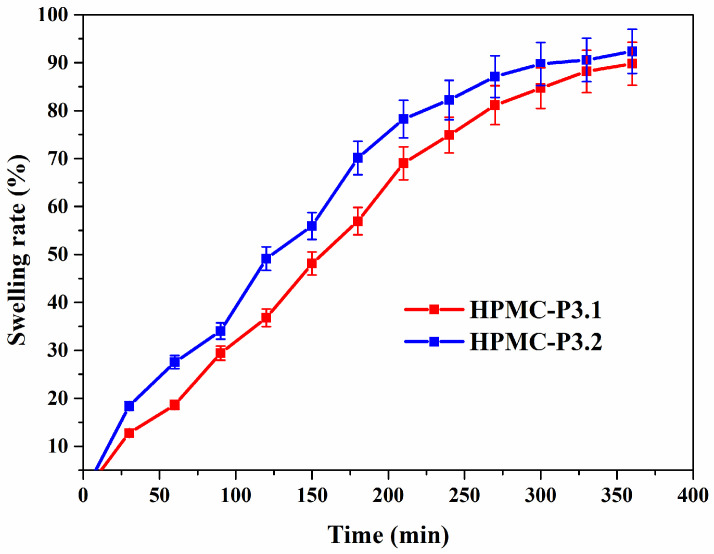
The swelling rate of HPMC-P3.1 (red line) and HPMC-P3.2 (blue line) over 6 h.

The swelling rate of the two samples (HPMC-P3.1 and HPMC-P3.2), tested over 6 h on 0.25 g of dried hydrogel, is represented in Figure 9 and the values are shown in Table 3.

The absorption capacity was almost the same for the two porphyrins incorporated into the HPMC hydrogel matrix, with a higher swelling rate in the first 200 min. Despite this fact, HPMC–P3.2 exhibited a slightly higher swelling degree (almost 92%) compared to HPMC–P3.1 (almost 88%) over 330 min. This behavior suggests that the incorporation of porphyrins may influence the microstructural organization of the HPMC hydrogel, leading to a polymer network capable of accommodating water molecules while maintaining a relatively compact structure. HPMC-P3.2 exhibited the highest swelling degree among the analyzed formulations. This behavior may be associated with the physicochemical characteristics of the incorporated porphyrin, particularly its polarity and affinity for the hydrophilic HPMC network. Such interactions could facilitate increased water penetration within the hydrogel structure, promoting matrix expansion and higher swelling values [56].

Based on the above results, a direct structure–properties correlation can be made: the incorporation of the porphyrins in the HPMC hydrogel network can induce an increased performance on the optical, rheological, and swelling properties, highlighting their potential use for further biomedical applications.

## 3. Conclusions

Novel porphyrin-incorporated HPMC hydrogel networks have been obtained by a simple solution mixing method through which the porphyrin molecule (P3.1 and P3.2) was embedded in the hydrogel matrix without any significant chemical modification. By maintaining the polymeric matrix at a constant, the study highlights a comprehensive evaluation of the influence of porphyrin structural features on the physicochemical and pharmacotechnical properties of the resulting hydrogel systems. The physical interactions, mostly by hydrogen bonding, were confirmed by FTIR analysis. The amorphous nature of the obtained hydrogel samples was established by X-ray diffraction, with small differences in the peak intensities. The uniform, homogeneous morphology demonstrated by AFM analysis was correlated with the UV–Vis and fluorescence data, which indicate that the porphyrins are in a monomeric state via preserving their optical properties. Further, rheological and swelling properties confirm the pseudoplastic behavior, a characteristic of hydrogels. The obtained P3.1 and P3.2 porphyrin-incorporated HPMC hydrogels show potential promise for diverse biomedical applications.

The obtained results demonstrate that porphyrin structure plays a significant role in the formulation features, including surface morphology, swelling behavior, rheological characteristics, and overall physicochemical stability. In particular, differences between P3.1 and P3.2 highlight the importance of substitution patterns and polarity in governing porphyrin–matrix interactions within the HPMC network. These findings support a structure–property relationship in which the photosensitizer, rather than the polymeric carrier, acts as the primary determinant of system behavior.

The use of HPMC as a validated and biocompatible hydrogel platform ensured formulation stability and reproducibility, while also allowing direct comparison between different porphyrinic systems under identical conditions. The study therefore provides new insights into porphyrin-centered design strategies for future topical photodynamic therapy formulation studies.

Future work will focus on extending this approach through in vivo evaluation to better correlate physicochemical properties with biological performance. Additionally, further studies on porphyrin structure–activity relationships may contribute to the rational design of next-generation photosensitizer-loaded hydrogel systems for dermatological applications.

## 4. Materials and Methods

### 4.1. Materials

The P3.1 and P3.2 porphyrins were prepared using the synthesis method previously detailed [15]. Hydroxypropyl methylcellulose (HPMC) and polyethylene Glycol 200 (PEG 200) were acquired from Sigma-Aldrich Chemie GmbH (Taufkirchen, Germany). All chemicals were of analytical grade and used as received without further purification. The chemicals were weighed using a Mettler Toledo AT261 balance (Columbus, OH, USA) (with a sensitivity of 0.01 mg).

### 4.2. Synthesis

**Scheme 2:**
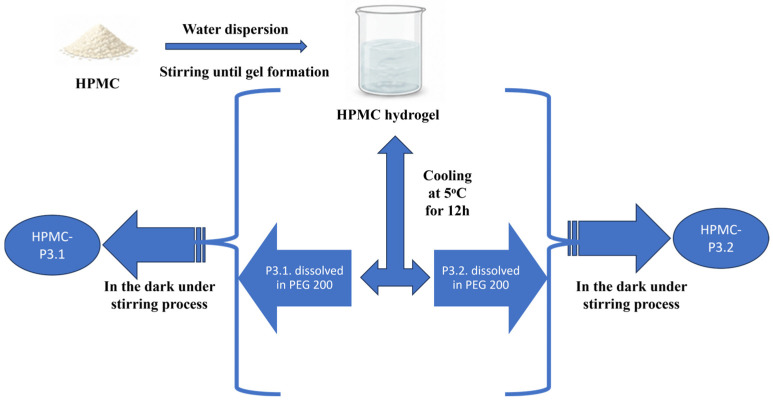
Schematic representation of HPMC-loaded porphyrin synthesis.

The synthesis of porphyrin–hydrogel samples was achieved using a simple, environmentally friendly solution mixing method (Scheme 2). The HPMC (100 mg/mL) was hydrated in double-distilled water under continuous stirring using a Heidolph MR 3001K magnetic stirrer (Schwabach, Germany) at 800 rpm at room temperature, until the hydrogel was completely formed. Next, by cooling at 5 °C for 12 h, the deaeration process was achieved. The obtained hydrogel was further used for the incorporation of porphyrins. The porphyrin compounds (P3.1 and P3.2) were separately dissolved in polyethylene glycol 200 (PEG) to obtain homogeneous solutions. The 10 mM porphyrin–PEG solutions were subsequently added to the 50 g HPMC hydrogel under continuous stirring at 750 rpm and in the dark, allowing the chromophores to be incorporated into the polymer network through physical entrapment and solvent-assisted mixing. The obtained HPMC–P3.1 and HPMC–P3.2 samples were further used for physicochemical investigations to elucidate the interface insights (as films of dried hydrogels or as hydrogels).

### 4.3. Characterization Methods

#### Pre-Dried Samples

FTIR spectra were performed on a JASCO FT/IR 4700 spectrophotometer (Tokyo, Japan) with a diamond ATR in the wavenumber range of 4000–400 cm^−1^, at a resolution of 4 cm^−1^. The XRD spectra were obtained by a Rigaku Ultima IV diffractometer (Rigaku Co., Tokyo, Japan), which works in parallel beam geometry and with CuKα radiation of 1.5406 Å (2θ = 5–60°, scanning speed of 2°/min, step size of 0.02°). Thermogravimetric analyses (TGA) were performed using a Mettler Toledo TGA/SDTA 851^e^ thermogravimeter (Mettler-Toledo, Greifensee, Switzerland). The analyses were carried out at a heating rate of 10 °C min^−1^, under 80 mL min^−1^ synthetic air flow.

Atomic force microscopy (AFM) analysis was performed in non-contact mode (XE100, Park System Corporate, Suwon, Republic of Korea), using PPP-NCHR probes (<10 nm tip radius, ~330 kHz resonant frequency, Nanosensors™). The decoupled scanner system ensured data acquisition without cross-talk artifacts. The data were processed in XEI software (v 1.8.0, Park Systems, Corporate, Suwon, Republic of Korea) to calculate the root mean square roughness (Rq) and the peak-to-valley parameter (Rpv), and the topographic images were accompanied by characteristic line-scans (surface profiles).

UV–Vis absorption spectra were recorded using a Specord 200 spectrophotometer (Analytik Jena, Jena, Germany) equipped with deuterium and halogen lamps with automatic switching for measurements in the 190–1100 nm range. All measurements were performed using 1 cm pathlength quartz cuvettes.

Fluorescence emission spectra were obtained using a JASCO FP-6500 spectrofluorometer (JASCO Co., Ltd., Kyoto, Japan). The excitation wavelength was fixed at 426 nm, corresponding to the Soret band maximum of the porphyrinic chromophores. Emission was recorded in the 600–750 nm range with a scan speed of 100 nm/min, a spectral bandwidth of 5 nm (excitation and emission), and a photomultiplier tube voltage (PMT gain) of 500 V. All fluorescence measurements were conducted in 10 mm pathlength quartz cuvettes.

Pharmacotechnical analysis.

(a) Hydrogel sample characterization

For determining the pH values of samples, the measurement was made by mixing 1 mL of distilled water at pH 6.5 ± 0.5 with 0.2 g of each sample, at room temperature for 5 min, using a CONSORT P601 pH-meter (CONSORT nv, Turnhout, Belgium).

Spreadability measurements were performed using 1 g of the sample placed on a glass plate. A glass plate of 153 g weight was applied on the surface. The diameter of the spread gel was measured after 2 min between each plate. Subsequently, additional weights of 50 g, 100 g, 150 g, 200 g, 250 g, 300 g, and 500 g were consecutively applied. The spreading area (mm^2^) of the hydrogel was calculated by using the measured diameter, using the following formula:

(1) $$S=\pi r^{2}$$

Rheology analysis was performed using 50 g of samples at 22 °C using a B-one Plus rotational viscometer (Lamy Rheology Instruments, Champagne du Mont d’Or, France), and rotation speeds between 50 rpm and 250 rpm and reverse, at 150 s between determinations.

(b) Dried film characterization

The thickness parameter of the dried films, obtained by drying at room temperature in Petri dishes for 48 h, was measured using a digital micrometer (Yato Trading Co., Ltd., Shanghai, China), with a measurement range between 0 and 25 mm and a resolution of 0.001 mm. The results are expressed as mean ± SD (n = 5).

Folding endurance tests on the 5 films of each formulation were repeatedly folded and rolled until the films were broken (approximately up to 300 times, at the same place). The results are reported as folding endurance values, which represent the recorded folding times.

The tensile strength and elongation behavior were evaluated using a digital LR 10K Plus tensile strength tester (Lloyd Instruments Ltd., West Sussex, UK), at a speed of 3 mm/s and a distance of 20 mm. The force at breakage (kg) was determined [57], which was subsequently used to obtain the *elongation* (%) and *tensile strength* (kg/mm^2^):

(2) tensile strengthkgmm2=force at breakagekgfilm thicknessmm×film widthmm

(3) $$elongation\left(\%\right)=\frac{increase\;in\;film\;length}{initial\;film\;length}\times 100$$

Moisture content was evaluated using the thermogravimetric method using an HR 73 Mettler-Toledo halogen humidity analyzer (Mettler Toledo GmbH, Greifensee, Switzerland). By weighing 0.25 g of the dried hydrogel every 30 min for a 6 h incubation period at 37 ± 1 °C on Petri dishes containing 1.5% agar gel, the *swelling ratio* (%) was determined, using Equation (4):

(4) $$swelling\;ratio\left(\%\right)=\frac{w_{t}-w_{i}}{w_{i}}\times 100$$

where *w_t_* is the sample weight at time *t* after the incubation period, and *w_i_* is the initial weight [58].

Statistical analysis.

All experiments were made in triplicate and are represented in tables as the mean ± SD (standard errors), and in figures by adding the error bars calculated as standard deviations.

## 5. Patents

Patent application 201900799: Rica Boscencu, Gina Manda, Laura Olariu, Ionela Victoria Neagoe, Radu Petre Socoteanu, Mihail Eugen Hinescu, Luis Filipe Vieira Ferreira, Antonio Cuadrado, Huveida Basaga, *Tetrapyrrolic derivative for antitumor photodynamic therapy and obtaining process*, published in RO-BOPI, 9 from 30 September 2020.

## Figures and Tables

**Table 1:** Spectral characteristics of P3.1 and P3.2 porphyrins into the HPMC polymer matrix.

	Absorption, λ_max_ (nm)					Emission, λ_max_ (nm)
	Soret Band	Qy (1,0)	Qy (0,0)	Qx (1,0)	Qx (0,0)	
HPMC-P3.1	403	500	532	572	657	650
HPMC-P3.2	405	498	531	571	655	651

**Table 2:** The organoleptic properties of HPMC–P3.1 and HPMC–P3.2.

Sample	Color	Appearance	Homogeneity	Consistency	pH	Density (g/mL)	Phase Separation
HPMC–P3.1	Translucent	Homogeneous, no air bubble	Very good	Good	7.0	1.09 ± 0.03	No phase separation
HPMC–P3.2	Translucent	Homogeneous, no air bubble	Very good	Good	7.0	1.14 ± 0.09	No phase separation

**Table 3:** The physicochemical parameters evaluated for HPMC-P3.1 and HPMC-P3.2 dried gels.

Variable	HPMC-P3.1	HPMC-P3.2
Thickness (mm)	0.059 ± 0.002	0.062 ± 0.003
Folding endurance value	>300	>300
Tensile strength (kg/mm^2^)	1.02 ± 0.06	1.16 ± 0.23
Elongation %	20.13 ± 1.48	26.05 ± 1.04
Swelling ratio (% after 6 h)	89.78 ± 0.31	92.37 ± 0.35

## Data Availability

The data presented in this study are available on request from the corresponding author.
